# Differences in Attitudes of Front-Line Clinicians, Healthcare Workers, and Non-Healthcare Workers Toward COVID-19 Safety Protocols

**DOI:** 10.7759/cureus.20936

**Published:** 2022-01-04

**Authors:** Megan Young, Riley Marotta, Isaac Lee, James Phan, Robin J Jacobs

**Affiliations:** 1 Dr. Kiran C. Patel College of Osteopathic Medicine, Nova Southeastern University, Fort Lauderdale, USA

**Keywords:** pandemic, social distancing, safety protocols, vaccines, mask wearing, perceptions, attitudes, coronavirus, sars-cov-2, covid-19

## Abstract

Background

The advent of the coronavirus disease 2019 (COVID-19) has generated varying opinions toward adhering to safety protocols among public health experts. With decreasing restrictions on public gatherings, lax protective behaviors, distortion of facts, and increasing availability of COVID-19 vaccines, response to public health guidelines vary greatly. Personal experiences with COVID-19, education, and work environment may influence decisions on safety recommendations and vaccination protocols among the public and healthcare professionals alike. To better understand how individuals process and make decisions regarding COVID-19 safety measures, this study investigated the attitudes among clinical and non-clinical healthcare workers and non-healthcare workers toward COVID-19 safety protocols.

Methodology

Cross-sectional data were collected from Florida residents using an online, 20-item anonymous questionnaire. Participants were recruited using the Florida Department of Health database for physician emails, social media, and snowball sampling strategies. The survey consisted of demographic items and questions regarding patient attitudes toward safety protocols for COVID-19 (e.g., likeliness to wear a mask in public despite state regulations being lifted, maintaining a distance of at least 6 feet between close friends and family, dining at restaurants/bars, gathering in groups larger than 10 people, getting a COVID-19 vaccine if one becomes available). Data were analyzed using one-way analysis of variance and chi-square test using SPSS version 27 (IBM Corp., Armonk, NY, USA).

Results

Of the 373 participants who completed the survey, 183 (49.1%) worked in the healthcare field, with 100 (28.6%) providing direct patient care. The rest (n = 190; 50.9%) reported that they do not work in the healthcare industry. Findings suggest that those working in healthcare with direct involvement in patient care were more likely to get the COVID-19 vaccine than those not working in healthcare. Additionally, those working in healthcare and providing direct patient care were more likely to think that masks were effective in reducing the spread of COVID-19 compared to those who worked in healthcare but did not provide direct patient care.

Conclusions

This study provides new insights into the attitudes of front-line clinicians, non-clinical healthcare workers, and the general population. Increasing health promotion efforts and debunking myths about COVID-19 may prove useful in mitigating the spread of the disease.

## Introduction

In March 2020, the World Health Organization (WHO) declared coronavirus disease 2019 (COVID-19) a global pandemic forcing individuals to adapt to a new set of rules, standards, and social practices for safety against transmission. Public health guidelines promoting social distancing and mask-wearing are essential in mitigating the spread of COVID-19. Many of the guidelines and regulations implemented by the government and public health departments were met with unrest and opposition [1,2]. The spread of COVID-19 has revealed varying opinions toward following safety protocols from public health experts. To fight the spread of COVID-19, acceptance and adherence to these public health guidelines are paramount.

COVID-19 personal safety measures

Mask-Wearing

Mask-wearing has been known to be an effective preventative measure to reduce the airborne transmission of viruses [3,4]. Despite this, some people are opposed to or are unable to wear masks for health reasons, increasing the likelihood of transmission and spreading COVID-19. In a study utilizing US-based tweets to assess opposition to mask-wearing, researchers identified 10% of 257,152 collected tweets mentioning opposition to wearing a mask based on personal opinions [5]. Reasons for opposition to mask-wearing included physical discomfort, lack of effectiveness, and inappropriate or unnecessary for certain circumstances [5]. Because mask-wearing is effective at limiting the spread of COVID-19, it is important to understand the reasons underlying the behaviors toward COVID-19 safety measures. For example, because men are typically more likely to engage in risk-taking behaviors, they are less likely to wear a mask. However, a meta-analysis found no significant difference in the physical use of face masks between men and women, but a difference in perceptions regarding face masks between genders was identified [6]. Men felt face masks infringed on their independence while females believed face masks to be more uncomfortable [6].

Social Distancing

Overall, women are more likely to engage in social distancing [7]. However, both sex and age have been reported to be significantly associated with adherence to social distancing, with female (vs. male) and older (vs. younger) participants reporting higher levels of social distancing [8]. Additionally, women tend to visit the doctor more often and care for themselves, which may be a driving factor in their choice to maintain distance, whereas men are seen as risk-takers who challenge norms [9,10]. In a recent study, Mahalik et al. investigated conformity to traditional masculinity norms and attitudes toward mask-wearing mediated by perceived benefits, perceived barriers (such as negative reactions from friends or co-workers), confidence in scientific experts, and empathy to individuals vulnerable to COVID-19 [11]. The study concluded that conforming to traditional masculinity norms contributes to believing that health-protecting behaviors are not beneficial, including mask-wearing. Additionally, those who conformed to traditional masculinity norms did not value scientific expertise [11].

Vaccination

In a study assessing attitudes of healthcare personnel toward receiving COVID-19 vaccination, willingness to receive vaccination varied based on their occupational role, with providers of direct care indicating that they would willingly receive the vaccine [12,13]. Direct medical providers are more inclined to get vaccinated compared to administrative staff and those who do not provide direct patient care [13]. Once COVID-19 vaccines became publicly available to US citizens over 16 years of age, a Centers for Disease Control and Prevention (CDC) survey of individuals aged 18-39 years examined attitudes toward vaccination and vaccination intent. The survey showed that younger adults (aged 18-24 years) were most likely to report being unsure about getting vaccinated or that they were not planning on getting vaccinated [14]. Adults in all age groups who reported having lower incomes, having lower education levels, lacking health insurance, and living outside of metropolitan areas reported the lowest desire to get vaccinated [13,14]. Moreover, women healthcare workers have lower vaccine acceptance rates than men and trans/non-binary individuals [13].

While there is some published literature on attitudes toward COVID-19 safety protocols among the public and certain subgroups, little is known about the differences between the general population and healthcare workers providing direct and non-direct patient care, as well as the differences in attitudes toward these measures between women and men. Hence, this study investigated the differences in attitudes, beliefs, and perceptions about COVID-19 safety protocols among individuals working in higher-risk, front-line health care, non-clinical healthcare settings, and those not employed in the healthcare sector.

## Materials and methods

A 20-item, anonymous quantitative survey developed by the researchers that included items on attitudes, beliefs, and perceptions about current and future COVID-19 safety protocols was distributed electronically via email and social media platforms.

Sample and recruitment

Data were collected in March 2021 from physicians, nurse practitioners, medical students, nurses, allied health professionals, and non-healthcare professionals in south Florida, United States (Miami-Dade, Broward, West Palm Beach counties) via emails, snowball sampling techniques, email listservs, social media platforms, and email addresses readily available on the Florida Department of Health website. The survey was offered online using REDCap, a web-based user-friendly electronic data capture tool for research studies. The questionnaire took between five and seven minutes to complete. The study was approved by the researchers’ university Institutional Review Board.

Assessment instrument

The survey was developed by the researchers and included items on attitudes, beliefs, and perceptions about current and future COVID-19 safety protocols. The instrument included eight demographic items (e.g., age, sex) and items regarding masks, social distancing, dining out, gathering in large groups, being vaccinated against COVID-19, risk factors for contracting COVID-19, type of treatment sought should one present with COVID-19 symptoms, and other demographic items (e.g., living with someone who is at risk, sex, age). The non-demographic survey items are presented in Table 1.

**Table 1:** Non-demographic survey items. *Response set: 1 = very unlikely, 2 = unlikely, 3 = neutral, 4 = likely, 5 = very likely. **Response set: 1 = very ineffective, 2 = ineffective, 3 = neutral, 4 = effective, 5 = very effective.

Likeliness of following COVID-19 safety protocols*
How likely are you to follow COVID-19 safety guidelines from medical and public health experts (e.g., Centers for Disease Control and Prevention [CDC])?
How likely are you to support a complete shutdown for 6 weeks if there is a second wave of COVID-19 cases?
How likely are you to wear a mask in public despite the state regulations being lifted?
How likely are you to use an N95 mask if more become available?
How likely are you to maintain a distance of at least 6 feet between close friends and family?
How likely are you to maintain a distance of at least 6 feet in public?
How likely are you to dine-in at restaurants/bars?
How likely are you to order carry-out from restaurants/bars?
How likely are you to gather in groups larger than 10 people?
How likely are you to get a COVID-19 vaccination if one becomes available?
Effectiveness of masks and social distancing measures**
How effective do you feel masks are at reducing the spread of COVID-19?
How effective do you feel 6 feet social distancing is at preventing the spread of COVID-19?

Data analysis

The Statistical Package for the Social Sciences (SPSS®) version 27 (IBM Corp., Armonk, NY, USA) computer software was used to analyze the data from this study. To maintain the accuracy of data entry, the data were cross-checked for errors such as out-of-range values and missing data as well as outliers. Questionnaires with more than one-third (33%) of missing data were excluded from data analysis. Distributional assumptions with univariate and multivariate normality statistics (tests for skewness and kurtosis) as well as by visual inspections of the empirical distributions were tested. Of the 377 surveys distributed, 373 complete surveys were collected (98.9% response rate). One-way analysis of variance (ANOVA) and chi-square test of independence were performed using SPSS version 27 statistical software.

## Results

Characteristics of the study sample

A total of 373 participants completed the survey. The mean age of the participants was 28.9 years (SD = 10.012; range = 8-74 years). Overall, the majority (n = 310; 83.1%) reported being female, 16.4% (n = 61) reported being male, 3% (n = 1) reported being non-binary, and 3% (n = 1) preferred not to answer. In total, 183 (49.1%) of the participants reported working in the healthcare field, with 100 (28.6%) providing direct patient care. The rest (n = 190; 50.9%) reported not working in the healthcare industry. Table 2 presents the summary statistics for the major study variables.

**Table 2:** Summary statistics of major study variables regarding the likelihood of engaging in protective COVID-19 behaviors. *Items with statistically significant mean differences between groups. COVID-19: coronavirus disease 2019; SD: standard deviation

	Provides direct patient care (n = 100)		Works in healthcare but no direct patient care (n = 83)		Does not work in healthcare (n = 190)		Total (N = 373)	
	M	SD	M	SD	M	SD	M	SD
Follow COVID-19 safety guidelines from medical and public health experts	4.58	0.638	4.54	0.786	4.50	0.788	4.53	0.749
Support a complete shutdown for 6 weeks if there is a second wave of COVID-19 cases	3.59	1.538	3.60	1.553	3.83	1.389	3.72	1.468
Wear a mask in public despite the state regulations being lifted	4.34	1.148	4.12	1.392	4.28	1.141	4.26	1.202
Use an N95 mask if more become available	3.61	1.348	3.80	1.359	3.71	1.355	3.70	1.352
Maintain a distance of at least 6 feet between close friends and family	3.17	1.334	3.07	1.386	3.05	1.486	3.09	1.422
Maintain a distance of at least 6 feet in public	4.46	0.797	4.37	0.996	4.49	0.775	4.46	0.834
Dine-in at restaurants/bars	3.47	1.262	3.45	1.399	3.48	1.284	3.47	1.301
Order carry-out from restaurants and/or bars	4.37	0.787	4.52	0.786	4.28	0.971	4.36	0.888
Gather in groups larger than 10 people	2.82	1.242	2.69	1.325	2.75	1.301	2.75	1.288
Get a COVID-19 vaccination if one becomes available*	4.13	1.361	3.92	1.579	3.54	1.638	3.78	1.572
Feel masks are effective at reducing the spread of COVID-19*	4.21	0.891	3.78	1.210	3.95	1.185	3.98	1.127
Feel 6 feet social distancing is effective at preventing the spread of COVID-19	4.00	1.044	3.65	1.301	3.84	1.127	3.84	1.150

Analysis of variance findings

To investigate the differences between groups regarding attitudes toward COVID-19 safety behaviors, one-way ANOVAs were conducted on each of the survey items on attitudes toward COVID-19 to compare the differences between groups (i.e., persons providing direct patient care, persons working in healthcare but not providing direct patient care, and persons not working in healthcare). Table 3 presents the significant ANOVA results regarding group differences in attitudes toward COVID-19 safety behaviors as the criteria.

**Table 3:** Significant ANOVA findings using differences between groups on attitudes toward COVID-19 safety behaviors. ANOVA: analysis of variance; COVID-19: coronavirus disease 2019; SD: standard deviation

	F(2,370-372)	P-value	ή^2^	Provides direct care (n = 110)		Works in healthcare (non-direct) (n = 83)		Does not work in healthcare (n = 190)	
Dependent variable				M	SD	M	SD	M	SD
Belief in mask effectiveness in reducing the spread of COVID-19	3.471	0.032	0.018	4.21	.891	3.78	1.210	3.95	1.185
Likely to get vaccinated against COVID-19	5.071	0.007	0.027	4.13	1.361	3.92	1.579	3.54	1.638

Effectiveness of Masks

ANOVA test showed that the effect of group membership on attitudes toward mask effectiveness was significant [F(2, 370) = 3.471, p = 0.032]. Post hoc comparisons using the Tukey honest significance test (HSD) test indicated that the mean score for the clinicians providing direct patient care on the item “How effective do you feel masks are at reducing the spread of COVID-19?” (using a five-point Likert scale indicating 1 = very ineffective and 5 = very effective) was significantly different than those working in healthcare but not providing direct patient care. However, persons not working in healthcare did not differ significantly from those providing direct patient care as well as those working in healthcare but not providing direct patient care.

Vaccination

ANOVA test showed that the effect of group membership on the likelihood of getting the vaccine once available was significant [F(2, 370) = 5.071, p = 0.007). Post hoc comparisons using the Tukey HSD test indicated that the mean score for the clinicians providing direct patient care on the item “How likely are you to get a COVID-19 vaccination if one becomes available?” (using a five-point Likert scale indicating 1 = very unlikely and 5 = very likely) was significantly different than those not working in healthcare. However, the persons working in healthcare but not providing direct patient care did not significantly differ from the clinicians providing direct patient care and persons not working in health care.

There were no statistically significant differences in mean scores between the three groups on the other 10 items.

## Discussion

Since the start of the COVID-19 pandemic, scientists, healthcare organizations, and companies globally have struggled to find ways to prevent and end the spread of the COVID-19 virus. From social distancing to wearing masks to developing a vaccine, the world rallied to find both practical and scientific means of protection. As more organizations and public health entities encourage and educate regarding various safety protocols, researchers around the world seek to identify individuals’ likelihood to adhere to the protocols, differences between individuals that may impact the likelihood of adhering to safety protocols, and the reasons for which individuals choose to adhere to COVID-19 safety protocols or not.

Of the 373 participants, 28.8% (n = 100) identified themselves as front-line clinicians, 22.3% (n = 83) identified as healthcare workers providing no direct care, and 50.9% (n = 190) identified as non-healthcare workers. Much of this study’s findings distinguish attitudes toward COVID-19 protocols based on three separate self-identified roles. Participants who identified as working in the healthcare field while also providing direct patient care are labeled as front-line clinicians. Examples of front-line clinicians include, but are not limited to, physicians, nurses, physician assistants, dentists, and pharmacists. Participants who identified as working in the healthcare field but not providing direct patient care were labeled as healthcare workers. Examples of healthcare workers include, but are not limited to, front office personnel at a physician’s office and hospital administrators. The last group of participants is those who did not identify as working within the healthcare field, which we labeled as non-healthcare workers. Examples of non-healthcare workers include bankers, business owners, and engineers.

Of the 10 items included in the survey, significant differences were only identified for the likelihood of wearing a mask and the likelihood of receiving COVID-19 vaccinations once available. Researchers infer that only these two items reveal a significant difference primarily due to the differences driven by media. Since the onset of COVID-19, there has been a constant battle between different social media outlets regarding the efficacy, risks, and benefits of different COVID-19 safety protocols, primarily mask-wearing and vaccinations. For this reason, stronger opinions are formed regarding these two issues based on the attitudes conveyed by different social media and news outlets. Consequently, researchers infer that, as a result of exposure, individuals are likely to have more significantly differing opinions towards mask-wearing and vaccinations as opposed to other COVID-19 safety guidelines.

Mask-wearing

Initially, it was thought there would be a significant difference in adherence to mask-wearing protocols between all three groups (front-line clinicians, non-healthcare workers, and healthcare workers). However, there were significant differences in willingness to wear masks only between front-line clinicians and healthcare workers. Not surprisingly, there was a significant difference in attitudes toward mask effectiveness between front-line clinicians and healthcare workers. On the other hand, there was no significant difference in attitudes toward mask effectiveness among non-healthcare workers, front-line clinicians, or healthcare workers. In conclusion, the collected data indicated there was no significant difference between non-healthcare workers, healthcare workers, and front-line clinicians and the likelihood of wearing a mask. These findings contradict the initial hypothesis as a significant difference was identified between front-line clinicians and healthcare workers, but no significant difference between front-line clinicians or healthcare and non-healthcare workers.

Previous studies have concluded that even a simple cloth mask can provide 50% efficacy in filtering small particles. Additionally, wearing a mask was found to decrease the distance traveled by droplets when coughing by at least 50% [4]. Another study specifically identified that surgical face masks provided a greater reduction in the spread of coronavirus RNA in aerosols. This study concluded that surgical face masks could prevent the transmission of COVID-19 from symptomatic individuals [3]. Consequently, the study concluded that one reason why front-line clinicians may be more willing to wear a mask compared to healthcare workers may be the simple explanation of accessibility [3]. Physicians presumably have increased access to surgical masks that have been proven to prevent the spread of COVID-19, which may make them more likely to utilize the mask compared to healthcare workers because front-line clinicians are aware of their scientifically proven efficacy.

Future studies should aim to identify the willingness to wear a mask based on self-identified occupational status or roles and availability of resources. For front-line clinicians working in large level I trauma centers, front-line clinicians likely have increased access to different types of masks compared to non-healthcare workers. Consequently, this availability may alter an individual’s willingness to wear a mask in public.

Vaccination

Similar to the hypothesis for self-identified roles and mask-wearing, we hypothesized that there would be a significant difference in the willingness to get the COVID-19 vaccine between front-line clinicians and non-healthcare workers as well as a difference between patient-facing healthcare workers and non-patient-facing healthcare workers. Because healthcare workers were the first group eligible to receive the vaccine, it was thought that their attitudes and beliefs toward vaccination may play a crucial role in public acceptance. As expected, there was a significant difference in the willingness to receive the COVID-19 vaccine between front-line clinicians and healthcare workers as front-line workers would have greater exposure, and thus a greater risk for contracting the virus.

The findings indicated differences in the likelihood of receiving the COVID-19 vaccine, when available, between front-line clinicians and non-healthcare workers. However, there was no significant difference in the likelihood to receive the COVID-19 vaccine between healthcare workers, front-line clinicians, or non-healthcare workers; moreover, no significant differences were found between healthcare workers and non-healthcare workers regarding getting vaccinated as previously thought. In this sample, the accessibility of the vaccine does not seem to contribute to vaccination status. The likelihood of receiving the vaccine may be based on other factors, including, but not limited to, personal preference, fear, or misinformation about the vaccine. Further studies could potentially explore the beliefs behind the COVID-19 vaccine and compare those who are pro-vaccine to those who are anti-vaccine.

Previous studies have demonstrated that front-line clinicians and healthcare workers base their decisions to adhere to safety protocols, such as receiving a vaccine, on published scientific literature discussing the efficacy and safety of safety protocols. Past studies have concluded that with a vaccine which is 100% effective, 70% of the population would require vaccination to reach herd immunity [13]. Combined with other studies reporting approximately 95% efficacy for the two primary COVID-19 vaccines in the United States, manufactured by Pfizer-BioNTech and Moderna, these findings clearly demonstrate the necessity for more publication of scientific literature-based protocols to advocate proper and effective safety protocols.

Additionally, there is a possibility that front-line clinicians may be more willing to receive the COVID-19 vaccine after directly interacting and witnessing the deleterious effects of COVID-19 in their patients. After seeing the illness first-hand and the devastating toll it takes on the health of those infected, it may be likely that front-line clinicians may be more willing to receive the vaccine. Future studies should aim to identify if there is a significant difference between front-line clinicians treating patients with COVID-19 versus front-line clinicians who do not interact with patients infected with COVID-19. Future investigations might consider identifying individuals who have been fully vaccinated with both doses of the COVID-19 vaccine from Pfizer-BioNTech and Moderna and their willingness to receive a third booster vaccination dose. Specifically, future studies should seek to identify if there is a significant difference in the willingness to receive the third boost dose and whether the third dose is approved by the Food and Drug Administration.

Implications for future research

Much of the apprehension to safety protocol adherence may be due to unsubstantiated, opinion-based information distributed through various social media outlets (e.g., Instagram, Facebook, TikTok). In addition to the recommendations for future studies, prospective studies should aim to identify if there is a difference between attitudes toward COVID-19 safety protocols and individuals’ sources of information. For example, a celebrity Instagram user can report adverse side effects after receiving the COVID-19 vaccine; however, they may neglect to report other health conditions and comorbidities that may have contributed to the side effects. Consequently, viewers and followers may read about the adverse side effects and decide they no longer want to receive the vaccine. It may be best to advocate the use of publications and literature that is supported by ample evidence, statistics, and analysis by appropriate entities (i.e., CDC and WHO), instead of personal experiences and opinions.

Updating the effectiveness of current safety protocols against new variants of the COVID-19 virus may be warranted. It may be important to investigate whether changes in the effectiveness against new variants will impact individuals’ attitudes toward COVID-19 safety protocols getting vaccinated or receiving booster shots.

Study limitations

This cross-sectional survey conducted in Florida cannot identify cause and effect and results cannot be generalized to other regions in the United States and globally. The survey was voluntary, raising the possibility of selection bias among those opting to participate. It is unclear whether those who feel more strongly, either positively or negatively, would be more likely to respond. Moreover, the survey was conducted at a single point in time during an ever-changing pandemic in which information, perceptions, and options were rapidly changing as new vaccines were approved and safety protocols evolved.

## Conclusions

This study provides new insight into the attitudes of clinicians, healthcare workers who are not patient-facing, and those who do not work in healthcare regarding COVID-19 safety protocols and vaccination. Data from this study suggest that those working in healthcare with direct patient care were more likely to get the COVID-19 vaccine than those not working in healthcare. Additionally, those working in healthcare and providing direct patient care were more likely to think that masks were effective in reducing the spread of COVID-19 compared to those who work in healthcare but do not provide direct patient care. Men and women perceive risk and engage in COVID-19 protective behaviors differently. Increasing public health and healthcare industry education may be useful regarding the vaccine and its effectiveness for those who do not work in healthcare or directly with patients.
